# Supplementary material for: Impacts of Proanthocyanidin Binding on Conformational and Functional Properties of Decolorized Highland Barley Protein
Source: Foods. 2023 Jan 19;12(3):481. doi: 10.3390/foods12030481 (PMC9914363; doi:10.3390/foods12030481)
Supplement: Supplementary file 1 [file foods-12-00481-s001.zip › foods-2138364-supplementary.pdf]

## Supplementary material

**Table S1** Effects of different addition level of PC on secondary structure of DHBP.

| | PC concentration | α-helix (%) | β-sheet (%) | β-turn (%) | Irregular curl(%) |
|---|---|---|---|---|---|
| DHBP- PC complex (pH=7) | 0.00 mmol/L | 18.89±0.16$^{a}$ | 26.25±0.07$^{d}$ | 22.90±0.01$^{a}$ | 31.86±0.23$^{a}$ |
| | 0.08 mmol/L | 17.98±0.22$^{b}$ | 27.07±0.25$^{c}$ | 23.03±0.03$^{a}$ | 31.92±0.50$^{a}$ |
| | 0.16 mmol/L | 17.69±0.19$^{b}$ | 27.39±0.19$^{bc}$ | 22.96±0.01$^{a}$ | 31.91±0.26$^{a}$ |
| | 0.32 mmol/L | 17.18±0.07$^{c}$ | 27.82±0.07$^{b}$ | 22.88±0.04$^{a}$ | 32.17±0.00$^{a}$ |
| | 0.64 mmol/L | 15.95±0.18$^{d}$ | 29.34±0.44$^{a}$ | 22.75±0.33$^{a}$ | 32.01±0.35$^{a}$ |
| DHBP- PC complex (pH=9) | 0.00 mmol/L | 14.24±0.37$^{a}$ | 28.59±0.41$^{b}$ | 23.40±0.20$^{a}$ | 33.77±0.24$^{b}$ |
| | 0.08 mmol/L | 13.63±0.15$^{b}$ | 28.89±0.62$^{ab}$ | 23.59±0.41$^{a}$ | 33.83±0.94$^{b}$ |
| | 0.16 mmol/L | 13.02±0.03$^{c}$ | 29.51±0.01$^{ab}$ | 23.62±0.05$^{a}$ | 33.84±0.07$^{b}$ |
| | 0.32 mmol/L | 12.53±0.14$^{c}$ | 29.56±0.00$^{a}$ | 23.28±0.04$^{a}$ | 34.58±0.26$^{ab}$ |
| | 0.64 mmol/L | 11.66±0.26$^{d}$ | 29.41±0.29$^{ab}$ | 23.33±0.05$^{a}$ | 35.60±0.36$^{a}$ |

Means followed by different letters indicate significant differences ($P$<0.05).

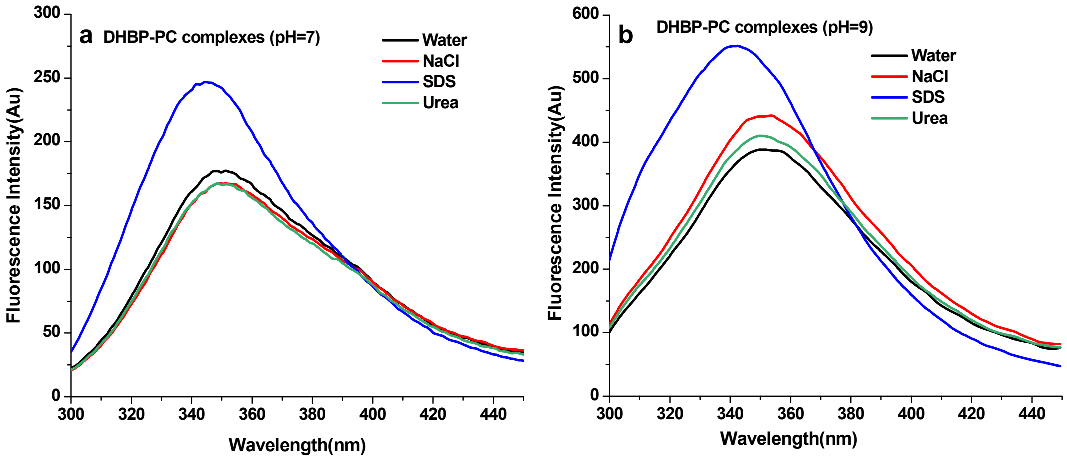


**Figure S1.** Effect of different blockers on DHBP-PC complexes.
